# Genetic determination of height-mediated mate choice

**DOI:** 10.1186/s13059-015-0833-8

**Published:** 2016-01-19

**Authors:** Albert Tenesa, Konrad Rawlik, Pau Navarro, Oriol Canela-Xandri

**Affiliations:** The Roslin Institute, The University of Edinburgh, Easter Bush Campus, Midlothian, EH25 9RG Scotland UK; MRC HGU at the MRC IGMM, University of Edinburgh, Western General Hospital, Crewe Road South, Edinburgh, EH4 2XU Scotland UK; Royal (Dick) School of Veterinary Studies, The University of Edinburgh, Easter Bush Campus, Midlothian, EH25 9RG Scotland UK

**Keywords:** Assortative mating, Genome structure, Missing heritability

## Abstract

**Background:**

Numerous studies have reported positive correlations among couples for height. This suggests that humans find individuals of similar height attractive. However, the answer to whether the choice of a mate with a similar phenotype is genetically or environmentally determined has been elusive.

**Results:**

Here we provide an estimate of the genetic contribution to height choice in mates in 13,068 genotyped couples. Using a mixed linear model we show that 4.1 % of the variation in the mate height choice is determined by a person’s own genotype, as expected in a model where one’s height determines the choice of mate height. Furthermore, the genotype of an individual predicts their partners’ height in an independent dataset of 15,437 individuals with 13 % accuracy, which is 64 % of the theoretical maximum achievable with a heritability of 0.041. Theoretical predictions suggest that approximately 5 % of the heritability of height is due to the positive covariance between allelic effects at different loci, which is caused by assortative mating. Hence, the coupling of alleles with similar effects could substantially contribute to the missing heritability of height.

**Conclusions:**

These estimates provide new insight into the mechanisms that govern mate choice in humans and warrant the search for the genetic causes of choice of mate height. They have important methodological implications and contribute to the missing heritability debate.

**Electronic supplementary material:**

The online version of this article (doi:10.1186/s13059-015-0833-8) contains supplementary material, which is available to authorized users.

## Background

The processes that lead humans to choose a particular mate and the extent to which these choices are governed by genes or environment have been widely debated. Here, we use 32,000 human couples and height as a model trait of human attractiveness to shed light onto these processes.

Height is a model quantitative trait that is determined by the interplay of large numbers of genetic and environmental factors. Narrow-sense heritability for height, which measures the relative importance of additive genetic factors and environmental factors in the expression of a trait, has been consistently estimated to be high, typically around 0.8 [1, 2]. Height has been associated with numerous diseases such as cancers [3], dementia death [4], and coronary artery disease [5]. However, all these associations, whether genetically or environmentally determined, are poorly understood.

The correlation in height between members of a couple is much larger than that expected by chance [6–10]. This indicates that humans tend to be attracted to mates that have a similar height to their own. Understanding this behaviour is sociologically important, but it is also biologically important. The consequences of assortative mating at the genetic level depend on the correlation among the breeding (or additive genetic) values of the mates. Assortative mating increases both the genetic and phenotypic variance compared to that observed in a random mating population [1] and plays a crucial role in shaping the genome structure of the population (i.e. how alleles are assorted) through increased coupling of alleles with positive or negative effects on the trait. Furthermore, because height is a highly polygenic trait [11], with hundreds of genes of small effect scattered across the genome contributing to its variation, it is possible that the build-up of directional linkage disequilibrium (LD) that arises from assortative mating impacts not only on the genetic architecture of height but also on that of many other complex traits. Despite its importance, the forces that drive mate choice for height and other traits are as yet unknown. To address this, we took height as a model trait and estimated to what degree mate height choice is genetically determined, and to what degree genes that contribute to one’s height are the same as those that affect individual preferences for mate height.

## Results and discussion

The UK Biobank [12] has genotyped ~30 % of its ~500,000 participants for an array that contains ~847,441 single nucleotide polymorphisms (SNPs). After employing stringent quality control (QC) criteria (see ‘Methods’), we extracted 13,068 self-reported and genetically inferred White-British (Additional file 1: Figure S1) male–female pairs that shared the same household address but were less related to each other than first cousins once removed, that is, with a coefficient of relationship (r) below 0.0625 (Additional file 1: Figure S2). Of these male–female pairs, ~92 % reported that they lived with their spouses, which is consistent with our hypothesis that these pairs were couples. We kept relatives (i.e. individuals with r > 0.0625) in our dataset providing they lived in different households (Additional file 1: Figure S3). Rare variants (those with minor allele frequency < 0.05) were removed from the analysis because they are known to distort the estimates of relatedness [13]. After removing possible outliers (see ‘Methods’), we modelled two phenotypes for each individual: the person’s own measured height and their partner’s measured height. The couples’ phenotypic correlation was 0.26 (95 % confidence interval [CI] 0.24, 0.27) (Additional file 1: Figure S4). We then adjusted for social and genetic population structure, correcting for the first 20 principal components (PCs) derived from an LD-pruned genomic relationship matrix (see ‘Methods’), age, gender, and Townsend deprivation index. The phenotypic correlation between couples remained high, at 0.23 (95 % CI 0.22, 0.24).

To estimate the contribution of genetic and environmental factors to variation in choice of mate height, we estimated relationships (Additional file 1: Figure S3) between the 26,136 individuals available [14] using the 318,852 autosomal SNPs that passed our QC protocol. We used a mixed linear model to estimate variance components [15]. To account for population and social structure, the analyses included the first 20 PCs, gender, age at recruitment, and Townsend deprivation index as fixed effects, and a genetic and an environmental (residual) random effect.

First, we used a univariate analysis to estimate to what degree attraction to a mate of similar height was explained by a person’s genotype. To that purpose, we treated the height of the partner as the person’s own trait (i.e. the choice of mate height). We estimated that the heritability of choice of mate height was 0.041 (standard error 0.014), which indicates that there is a significant genetic component for choice of mate height in humans. This is consistent with a model where mate selection for height is driven by one’s own height (see ‘Methods’).

We then asked whether the genetic determinants of choice of mate height were shared with the genetic determinants of a person’s own height. To answer this question, we treated the height of the partner as a phenotype of an individual and used a bivariate analysis to estimate the genetic and environmental correlation between the two traits. A genetic correlation equal to zero would imply that one’s own height and the choice of mate by height are not affected by the same genetic variants or that there is no directional pleiotropy, whilst a genetic correlation of one would imply that the two traits share the same genetic determinants, working in the same direction. Similarly, a non-zero environmental correlation would imply that the factors that affect the environmental and non-additive genetic deviations are at least partly shared between the two traits. The bivariate analysis (Table 1) performed using all available autosomal SNPs revealed that additive genetic factors explained 60 % and 3.6 % of the phenotypic variation for height and choice of mate height, respectively. These estimates are consistent with the estimates obtained in the univariate analysis. By analysing both traits jointly, we also demonstrated that 89 % of the genetic variation that affects height and choice of mate height is shared. Overall, this indicates that there is an innate preference for partners of similar height. To investigate this further we removed all related individuals (r > 0.0625) and performed two genome-wide association studies, one for height and one for choice of mate height. The correlation among estimated SNP effects was 0.25 (Additional file 1: Figure S5), which supports the hypothesis that height and choice of mate height share a substantial number of contributing loci and that alleles that increase height also, on average, increase attraction for increased height.

**Table 1:** Bivariate analysis of height (h^2^_Height_) and the choice of mate by height (h^2^_Height choice_) in White-British

	Estimate	Standard error
*h* ^*2*^_*Height*_	0.599	0.015
*h* ^*2*^_*Height choice*_	0.036	0.013
*r*_*G*_	0.887	0.148
*r*_*E*_	0.163	0.017
*r*_*P*_	0.232	0.006

rG is the genetic correlation, rE is the environmental correlation, rP is the phenotypic correlation

To strengthen the evidence for this hypothesis, we estimated, using genetic marker information and a univariate mixed-linear model (see ‘Methods’), the additive genetic effect (also known as breeding value in the quantitative genetics literature) for the height of individuals whose partner had not been genotyped, but for whom we had information on height. We reasoned that if the genetic correlation between height and choice of mate height was high, then we would be able to predict the height of one of the partners from the additive genetic effect (i.e. breeding value) for the height of the other partner. The correlation between the additive genetic effect for one’s own height and one’s partner’s height phenotype (i.e. the accuracy of prediction) was 0.13 (*P* = 7.55 × 10^−59^), that is, 64 % of the maximum expected correlation; the expected maximum correlation between the additive genetic effect for choice of mate height and phenotype for choice of mate height being 0.2, the square root of the heritability of choice of mate height.

**Fig. 1:**
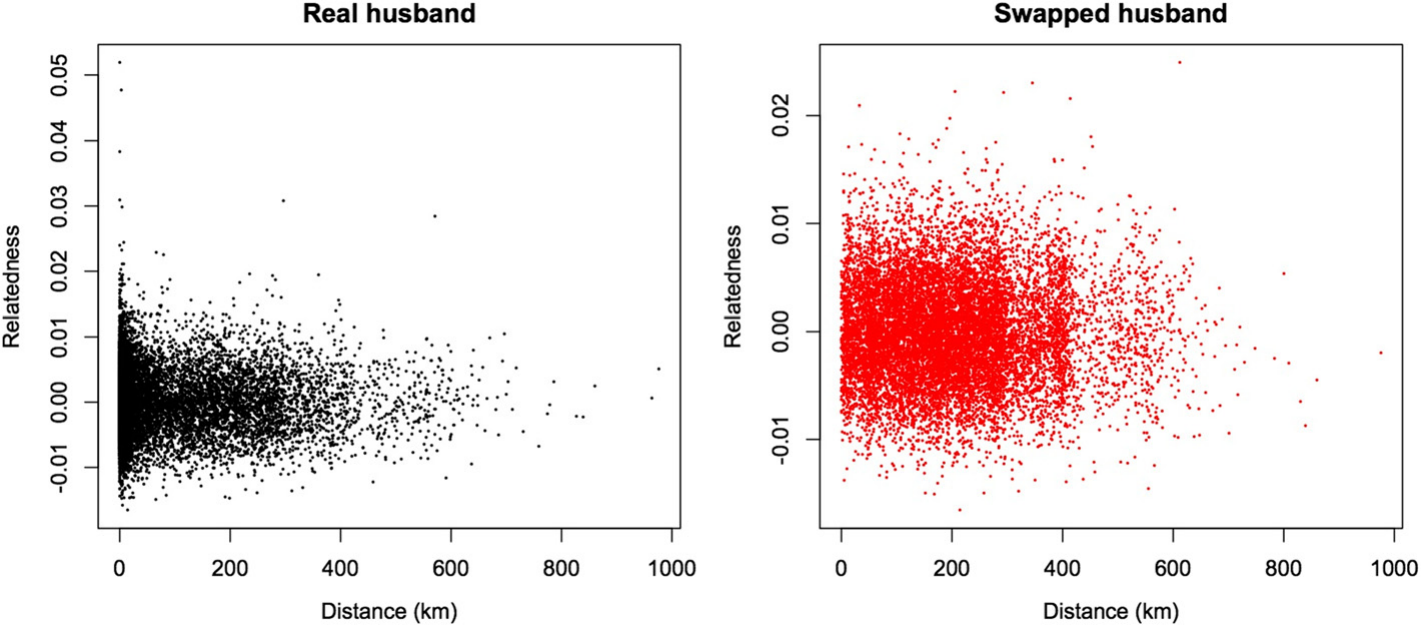
Correlation between distance of birthplaces and relatedness. The regression coefficient of relatedness on distance (m) was −7.9 × 10^−10^(*P* = 0.026) and −4.9 × 10^−10^ (*P* = 0.134), for the real husband and swapped husband, respectively

**Fig. 2:**
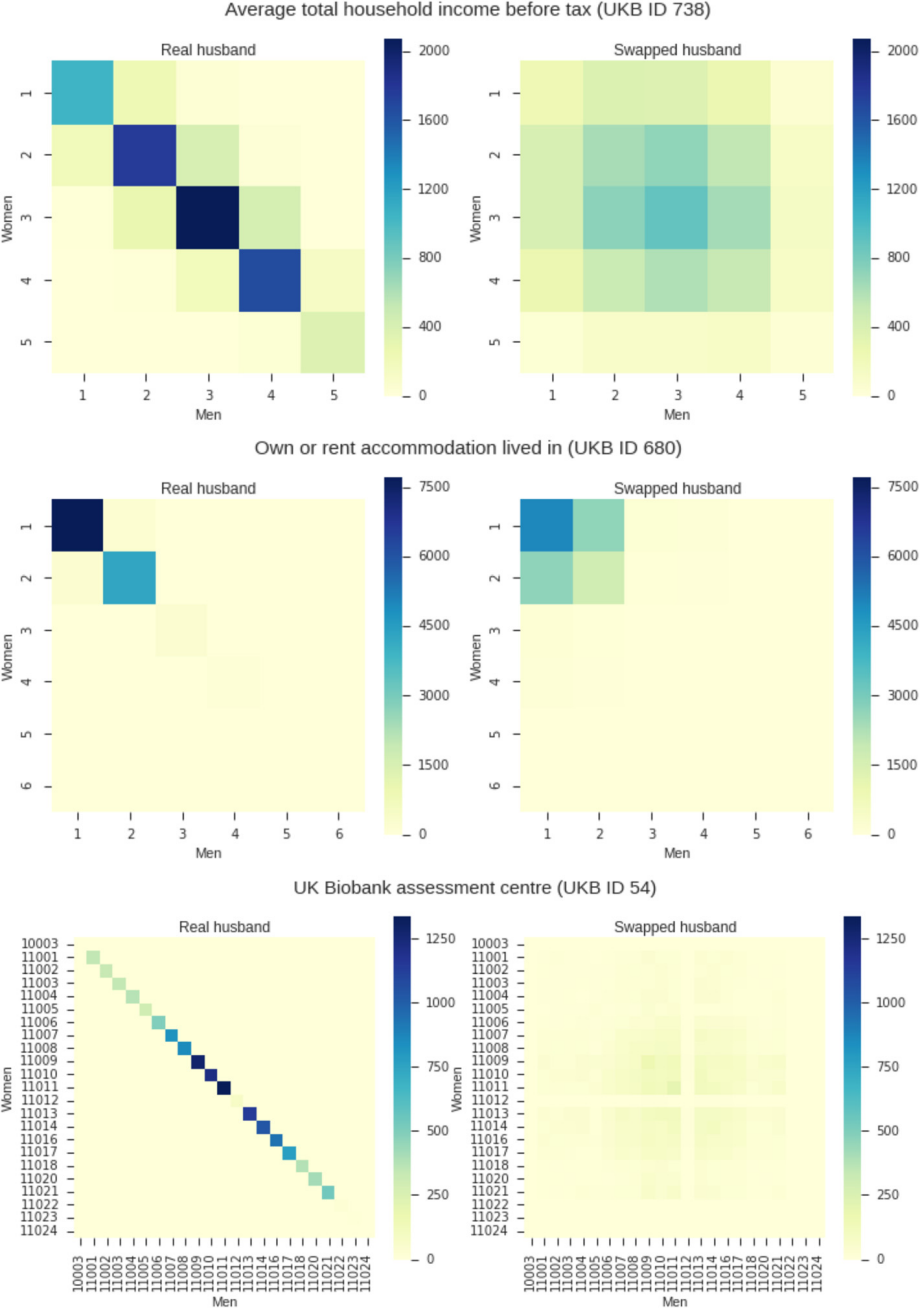
Histograms of covariates for the observed (*left*) and swapped (*right*) husband. The UK Biobank ID of the individual variables is provided in the titles, and axis are labelled by the UK Biobank coding (values indicating missing information were excluded). The number of individuals in each category is colour coded

The genetic consequences of assortative mating depend on whether the primary cause of assortment among partners is phenotypic (e.g. tall people are attracted to tall people), genetic (e.g. matings are within differentiated ethnic groups) or environmental (e.g. matings are with socially homologous groups). Primary genetic or environmental correlations arise when mating occurs within groups that are either genetically or environmentally differentiated. We argue that for human height the primary source of partner similarity is phenotypic, rather than caused by genetic or environmental structure within the population. We believe that the observed correlation in height between partners is not an artefact of mating within groups or populations that are genetically differentiated, because our analyses were adjusted for the first 20 PCs and because, for mixed-origin couples (those for which a partner is classified as White-British and the other as non White-British), we observed similar heritabilities to those of White-British couples for both height and mate’s height (Additional file 1: Table S1). In addition, we performed an analysis following a permutation approach that, whilst maintaining a height-associated mating structure, removed any genetic (Fig. 1) and environmental (Fig. 2) within-pair structure due to assortment based on alternative factors like geography, age or socio-economic status (see ‘Methods’). Specifically, we swapped the male partners amongst pairs of couples with similar phenotypes for both individuals. The results of this analysis (Additional file 1: Table S2) were practically identical to the results obtained for the original data, indicating that the genetic or environmental structure of the population is not driving the correlation between mates (Additional file 1: Table S3 and Fig. 2).

Assortative mating by phenotype results in an increase of additive genetic variance, and possibly heritability. The correlation of breeding values of the mates was estimated to be 0.2 (*P* = 1.07 × 10^−86^), which is consistent with the primary source of assortment being the phenotype. Moreover, we can use theoretical predictions to estimate what the heritability of height would have been before assortative mating started. If we assume a heritability for height of 0.8 under assortative mating, then we estimate that the heritability under random mating would be 0.76. That is, continuous assortative mating is expected to lead to an increase of heritability for height of ~5 %, which is due to how alleles are assorted in the population under assortative mating and not to additional genetic variants segregating in the population (‘Methods’).

Previous studies aimed at understanding assortative mating for height have not investigated the role that genetic variation plays in mate choice or mate attraction. Our results show that the observed attraction for mates of a similar height phenotype is partly genetically determined and this genetic variation is largely shared with the genetic variation that determines variation in individual height. Assortative mating generates directional LD between alleles that increase or decrease the trait values, that is, combinations of alleles that increase or decrease height tend to be at a higher frequency than expected by chance. This leads to an increase in additive genetic variance, especially for traits under strong assortative mating [16]. These findings offer the opportunity to search for the genetic variants and mechanisms that determine individual preferences for mate height, as well as other traits that govern human sexual attraction. Our results also have important implications with regards to models needed to analyse cohorts containing related individuals and couples, as well as contribute to solve the ‘missing heritability mystery’.

## Conclusions

We show that genetic variation influences the choice of partner. The genetic correlation between height and the preference for a partner with similar height is 0.89, which indicates that genes affecting individual preferences for height and one’s own height are largely shared. Furthermore, we used this observation to predict the height of the chosen partner from the person’s genotype with an accuracy equal to 64 % of the theoretical maximum. Finally, theoretical models predict that ~5 % of the heritability of height is due to non-random assortment of alleles caused by assortative mating and not to existing genetic variants, hence being a substantial contributor to the missing heritability of height.

## Methods

### Genotype quality control

We obtained data for 152,736 individuals genotyped in phase 1 of the UK Biobank genotyping programme. These comprise 49,979 individuals selected as part of the BiLEVE study based on their lung function phenotypes [17], 102,750 individuals selected at random amongst the remaining UK Biobank participants, and seven individuals with missing information, who were removed from further analyses. Genotypes were assayed using two platforms, the Affymetrix UK BiLEVE Axiom array for the BiLEVE cohort and the Affymetrix UK Biobank Axiom array [18] for all remaining individuals. The data consist of genotype calls for 847,441 markers, approximately 95 % of which are present on both genotyping platforms employed. Details regarding the genotyping procedure and genotype calling protocols are provided elsewhere [19] and in the following we only summarise any subsequent QC and processing performed. We excluded individual markers from further analysis if they were multi-allelic, their overall missingness rate exceeded 2 %, or if they exhibited a strong platform-specific missingness bias (Fisher’s exact test, *P* < 10^−100^). Individuals were excluded from further analysis if they exhibited excess heterozygosity, as identified by UK Biobank internal QC procedures [19], if their missingness rate exceeded 5 %, or if their self-reported sex did not match genetic sex estimated from X chromosome inbreeding coefficients. These criteria resulted in a reduced dataset of 151,532 individuals. Finally, we filtered out markers exhibiting a departure from Hardy–Weinberg equilibrium (*P* < 10^−50^) or with minor allele frequency below 0.05 within the subset of couples identified as ethnically White-British as described below, which left 318,852 SNPs for analysis. The genotype QC was performed using PLINK [20].

### Ethnicity

The UK Biobank cohort includes individuals of diverse ethnicities that may confound analyses. We therefore identified a core subset of 123,847 individuals of White-British ethnicity by combining self-reported and genotype information. Specifically, we performed a principal components analysis (PCA) of all individuals passing genotypic QC using an LD-pruned set of 99,101 autosomal markers (http://biobank.ctsu.ox.ac.uk/crystal/refer.cgi?id=149744) that passed our SNP QC protocol. Amongst individuals who self-reported their ethnicity as White-British, we then retained individuals for whom the projections onto the leading 20 genomic PCs fell within 3 standard deviations (SD) of the mean.

### Couples

Using household sharing information we identified a set of 105,381 households with exactly two members in the cohort that we considered to be couples. For 94,651 out of those 105,381 households, both residents report the same household size and relationship to other household members to be ‘Husband, wife or partner’ or both ‘Husband, wife or partner’ and ‘Son and/or daughter (include step-children)’. Hence, for ~90 % of the pairs we have additional confirmatory information that these were couples. Our univariate and bivariate analyses included only those couples whose coefficient of relatedness (r) was less than 0.0625, of which only seven pairs had r > 0.025.

Of those 105,381 identified couples, we used 13,068 White-British couples and 3,726 mixed-race couples (where one member of the couple was classified as White-British and the other as non White-British) that had been genotyped in phase 1. The bulk of the analyses in the paper were performed on the 13,068 White-British couples. Our predictions of the partner’s height based on the individual genotype were performed using 15,437 couples where only one of the partners had been genotyped in phase 1. For this, we predicted the total additive effect of height for the person genotyped and estimated the correlation (i.e. the prediction accuracy) of that ‘polygenic score’ with the height of their partner. In this case, and because we could not confirm that the partners were unrelated using genotypes, we set additional filtering criteria. We used only pairs where both individuals were self-reported White-British; the genotyped person was classified as White-British based on genotype; individuals reported different ages for one or both parents; and individuals had an age difference of less than 10 years, were of opposite gender, and reported to live with their partner or partner and children.

### Phenotype quality control

We defined outliers as males and females that were more than 3 SD from their gender mean, and removed them from the analyses.

### Model fitting

The PCA and all mixed linear models were fitted using DISSECT [21], a software tool designed to perform genomic analyses on large volumes of data in a high performance computing (HPC) environment without the need to perform mathematical approximations. DISSECT is an open access software that can be downloaded from our dedicated web site (http://www.dissect.ed.ac.uk) under a GNU GPL v3 license. The availability of the software and access to the UK National Supercomputer (ARCHER) allowed us to fit these computationally intense analyses to the large dataset.

### Univariate mixed linear model

We fitted the following univariate mixed linear model:

$$ {y}_i=\mu +{\displaystyle \sum_{l=1}^L{x}_{il}{\beta}_l+{\displaystyle \sum_{j=1}^M{z}_{ij}{a}_j+{e}_i,}} $$

where *μ* is the mean term and *e*_*i*_ the residual for individual *i. L* is the number of fixed effects, *x*_*il*_ being the value for fixed effect *l* for individual *i* and *β*_*l*_ the estimated effect for *l. M* is the number of markers and *z*_*ij*_ is the standardised genotype of individual *i* at marker *j*. The vector of random SNP effects **a** is distributed as N(0, **I***σ*_*u*_^2^). The phenotypic variance-covariance matrix is var(**y**) = **V** = **ZZ**^T^σ_*u*_^2^ + **I**σ_*e*_^2^. The SNP effects are estimated using the equation [16]:

$$ \mathbf{a}={\upsigma}_u^2{\mathbf{Z}}^T{\mathbf{V}}^{\hbox{-} 1}\left(\mathbf{y}\mathbf{\hbox{-}}\boldsymbol{\upmu} \mathbf{\hbox{-}}\mathbf{X}\boldsymbol{\upbeta} \right). $$

Because ∑_*j=1*_^*M*^*z*_*ij*_*a*_*j*_is the total additive genetic effect (*g*_*i*_) for individual *i*, this model can also be expressed as,

$$ {y}_i=\mu +{\displaystyle \sum_{l=1}^L{x}_{il}{\beta}_l+{g}_i+{e}_i.} $$

In this model, the vector of genetic effects **g** is distributed as N(0, **A**σ_*g*_^2^). Where **A** is the genetic relationship matrix and *σ*_*g*_^2^ = *Mσ*_*u*_^2^. Accordingly, the total phenotypic variance-covariance matrix is var(**y**) = **V** = **A**σ_*g*_^2^ + **I**σ_*e*_^2^. From the equivalence between these two models, DISSECT can estimate the total additive effect from the equation:

$$ \mathbf{g}={\upsigma}_{\mathrm{g}}^2\mathbf{A}{\mathbf{V}}^{\hbox{-} 1}\left(\mathbf{y}\mathbf{\hbox{-}}\boldsymbol{\upmu} \mathbf{\hbox{-}}\mathbf{X}\boldsymbol{\upbeta} \right). $$

DISSECT estimates *σ*_*g*_^2^ and *σ*_*e*_^2^ using the expectation maximization (EM) method for the first step [16], followed by AI REML method steps [22, 23].

### Bivariate mixed linear model

We fitted the following bivariate mixed linear model [24]:

$$ \mathbf{y}=\left(\begin{array}{c}\hfill {\mathbf{y}}_1\hfill \\ {}\hfill {\mathbf{y}}_2\hfill \end{array}\right)=\left(\begin{array}{c}\hfill {\boldsymbol{\upmu}}_1\hfill \\ {}\hfill {\boldsymbol{\upmu}}_2\hfill \end{array}\right)+\left(\begin{array}{cc}\hfill {\mathbf{X}}_1\hfill & \hfill 0\hfill \\ {}\hfill 0\hfill & \hfill {\mathbf{X}}_2\hfill \end{array}\right)\left(\begin{array}{c}\hfill {\boldsymbol{\upbeta}}_1\hfill \\ {}\hfill {\boldsymbol{\upbeta}}_2\hfill \end{array}\right)+\left(\begin{array}{c}\hfill {\mathbf{g}}_1\hfill \\ {}\hfill {\mathbf{g}}_2\hfill \end{array}\right)+\left(\begin{array}{c}\hfill {\mathbf{e}}_1\hfill \\ {}\hfill {\mathbf{e}}_2\hfill \end{array}\right), $$

where ***μ***_*i*_ is a vector of equal mean terms and ***e***_*i*_ the vector of residuals for the trait *i*. ***X***_*i*_ is the incidence matrix of the fixed effects ***β***_*i*_ for the trait *i*. ***g***_*i*_ is the vector of the individuals' genetic effects for the trait *i* with covariance matrix:

$$ \mathrm{v}\mathrm{a}\mathrm{r}\left(\begin{array}{c}\hfill {\mathbf{g}}_1\hfill \\ {}\hfill {\mathbf{g}}_2\hfill \end{array}\right)=\left(\begin{array}{cc}\hfill {\mathbf{A}}_1{\upsigma}_{g_1}^2\hfill & \hfill {\mathbf{A}}_{12}{\upsigma}_{g_1{g}_2}\hfill \\ {}\hfill {\mathbf{A}}_{12}^{\mathrm{T}}{\upsigma}_{g_1{g}_2}\hfill & \hfill {\mathbf{A}}_2{\upsigma}_{g_2}^2\hfill \end{array}\right), $$

where **A**_*i*_ is the genetic relationship matrix between the individuals measured for trait *i* and **A**_*ij*_ the genetic relationship matrix between the individuals measured for trait *i* and trait *j.*${\sigma}_{g_1}^2,{\sigma}_{g_2}^2,\kern0.5em \mathrm{and}\kern0.5em {\sigma}_{g_1{g}_2}$ are the genetic variance for trait 1, the genetic variance for trait 2 and the genetic covariance between the two traits, respectively. The phenotypic covariance matrix (**V**) is,

$$ \mathrm{v}\mathrm{a}\mathrm{r}\left(\mathbf{y}\right)=\mathbf{V}=\left(\begin{array}{cc}\hfill {\mathbf{A}}_1{\upsigma}_{g_1}^2\hfill & \hfill {\mathbf{A}}_{12}{\upsigma}_{g_1{g}_2}\hfill \\ {}\hfill {\mathbf{A}}_{12}^{\mathrm{T}}{\upsigma}_{g_1{g}_2}\hfill & \hfill {\mathbf{A}}_2{\upsigma}_{g_2}^2\hfill \end{array}\right)+\left(\begin{array}{cc}\hfill \mathbf{I}{\upsigma}_{{\mathrm{e}}_1}^2\hfill & \hfill {\mathbf{I}}_{12}{\upsigma}_{{\mathrm{e}}_1{\mathrm{e}}_2}\hfill \\ {}\hfill {\mathbf{I}}_{12}^{\mathrm{T}}{\upsigma}_{{\mathrm{e}}_1{\mathrm{e}}_2}\hfill & \hfill \mathbf{I}{\upsigma}_{{\mathrm{e}}_2}^2\hfill \end{array}\right), $$

where ${\sigma}_{e_1}^2,{\sigma}_{e_2}^2,\kern0.5em \mathrm{and}\kern0.5em {\sigma}_{e_1e{}_2}$ are the environmental variance for trait 1, the environmental variance for trait 2 and the environmental covariance between the two traits, respectively. **I** is the identity matrix and **I**_12_ is a matrix where the elements in row *i* and column *j* are 1 if the individual *i* for the trait 1 is the same than the individual *j* of the trait 2 and 0 otherwise. As in the univariate case, DISSECT fits the variances and covariances using the expectation maximization (EM) method for the first step [16], followed by AI REML method steps [22, 23].

### Estimation of heritability before assortative mating

The heritability of height, before the population started assortative mating and reached an equilibrium (*h*_0_^2^), was estimated as ${h}_0^2={h}^2\left[\frac{1-m}{1+m{h}^2}\right]$, where *h*^2^ is the current *h*^2^ (assumed to be 0.8), and *m* is the correlation of breeding values among mates [25].

### Permutation based analysis

We swapped the male individuals between pairs of couples where both the male and female where of similar height. This was achieved by ordering couples by both female and male heights, and swapping the male individual between pairs of successive couples, i.e., male 1 with 2, 3 with 4, and so on.

We first confirmed that this approach removed the genetic structure arising due to assortment by geography by regressing a couple’s relatedness on the distance of birthplaces (Fig. 1). Furthermore, we examined some of the available covariates that we thought might be related to social and geographical structure and which individually explained more than 0.5 % of variation in height, excluding covariates specific to only one sex and ‘Comparative height size at age 10’. For continuous covariates we computed the between partner Pearson’s correlations for the observed and permuted couples. For categorical covariates we examined the mutual information between the partner’s covariates as a measure of their dependence, computing the *p* value for the null hypothesis of zero mutual information from 1,000 permutations of the female covariates. We found that our permutation approach severely reduced dependence between partners in all variables (Additional file 1: Table S3). Although we found statistically significant associations for all but one variable in the observed data, after permutation, associations for all but three variables where not significantly different from zero. This was so despite the large sample size, which would allow us to detect even very small associations. For the three variables with statistically significant associations in the permuted data, examination of histograms (Fig. 2) of the observed and permuted data did not suggest the presence of any strong remaining structure.

### Expected heritability of assortative mating driven by phenotype

Let us assume a standardised phenotype (*P*) which follows the standard additive genetic model, i.e., *P*_*i*_ = *A*_*i*_ + *E*_*i*_, where *A*_*i*_ and *E*_*i*_ are the additive genetic effect and environmental component for individual *i*, respectively. If *P* drives mate selection the phenotype of the partner *m*(*i*) for a given individual *i* can be expressed as *P*_*m*(*i*)_ = *bP*_*i*_ + *E*_*m*(*i*)_. However under the assumption of an ante-dependence model *A*_*i*_ → *P*_*i*_ → *P*_*m*(*i*)_ for selection, where the additive genetic effect *A*_*i*_ influences the phenotype *P*_*i*_ which in turn influences the choice of *i*’s partner’s phenotype, we have *cov*(*A*_*i*_, *P*_*m*(*i*)_) = 0. Hence

$$ \operatorname{var}\left({P}_{m(i)}\right)={b}^2\operatorname{var}\left({A}_i\right)+{b}^2\operatorname{var}\left({E}_i\right)+\operatorname{var}\left({E}_{m(i)}\right) $$

with the genetic component being *b*^2^*var*(*A*_*i*_) = *b*^2^*h*_*P*_^2^*var*(*P*_*i*_) where *h*_*P*_^2^ is the heritability of *P*. Since for a standardised phenotype *b* = *r*_*p*_ where *r*_*p*_ is the phenotypic correlation and *var*(*P*_*i*_) = *var*(*P*_*m*(*i*)_), we have that ${h_{P_m}}^2={r}_p^2{h}_P^2$ is the expected heritability of partner’s phenotype.

Now assuming a heritability for height of *h*^2^ = 0.8 and *r*_*p*_ = 0.26, the expected heritability for choice of mate’s height is 0.043.

### Ethical approval

The use of the UK Biobank dataset falls within the study’s ethical approval from the North West Medical Research Ethics Committee (Reference 11/NW/0382).

### Availability of supporting data

The data can be accessed through the UK Biobank (http://www.ukbiobank.ac.uk).

## Additional file

Additional file 1:
**The following additional data are available with the online version of this paper.** Additional data file 1 contains five figures and three tables. Figure S1 plots the first (PCA1) and second (PCA2) principal components for phase 1 of the UK Biobank. Figure S2 shows a histogram of the relationships among couples. Figure S3 shows a histogram of relationships used in the univariate and bivariate analyses. Figure S4 shows the correlation of height for couples and correlation of height adjusted for gender, age, Townsend deprivation index and first 20 PCs. Figure S5 shows the correlation among genome-wide association studies estimated effects for own height and partner’s height choice. Table S1 shows the results from the bivariate analysis of height (h^2^_Height_) and the choice of mate by height (h^2^_Height choice_) in mixed couples. Table S2 shows the results from the bivariate analysis of height (h^2^_Height_) and choice of mate by height (h^2^_Height choice_) for White-British couples where partners have been swapped. Table S3 shows the correlations between observed partners and swapped partners for covariates explaining individually more than 0.5 % of variation in height. (DOCX 1010 kb)
