# Erratum to: Genetic determination of height-mediated mate choice

**DOI:** 10.1186/s13059-016-0894-3

**Published:** 2016-02-11

**Authors:** Albert Tenesa, Konrad Rawlik, Pau Navarro, Oriol Canela-Xandri

**Affiliations:** 1 The Roslin Institute, The University of Edinburgh, Easter Bush Campus, Midlothian, EH25 9RG Scotland UK; 2 MRC HGU at the MRC IGMM, University of Edinburgh, Western General Hospital, Crewe Road South, Edinburgh, EH4 2XU Scotland UK; 3 Royal (Dick) School of Veterinary Studies, The University of Edinburgh, Easter Bush Campus, Midlothian, EH25 9RG Scotland UK

The published version of this article states an incorrect publication year of 2015. The correct date of publication is 19^th^ January 2016. The publisher apologises for this error.
