# Supplementary material for: Genetic determination of height-mediated mate choice
Source: Genome Biol. 2016 Jan 19;16:269. doi: 10.1186/s13059-015-0833-8 (PMC4717574; doi:10.1186/s13059-015-0833-8)
Supplement: Additional file 1: — The following additional data are available with the online version of this paper. Additional data file 1 contains five figures and three tables. Figure S1 plots the first (PCA1) and second (PCA2) principal components for phase 1 of the UK Biobank. Figure S2 shows a histogram of the relationships among couples. Figure S3 shows a histogram of relationships used in the univariate and bivariate analyses. Figure S4 shows the correlation of height for couples and correlation of height adjusted for gender, age, Townsend deprivation index and first 20 PCs. Figure S5 shows the correlation among genome-wide association studies estimated effects for own height and partner’s height choice. Table S1 shows the results from the bivariate analysis of height (h2 Height) and the choice of mate by height (h2 Height choice) in mixed couples. Table S2 shows the results from the bivariate analysis of height (h2 Height) and choice of mate by height (h2 Height choice) for White-British couples where partners have been swapped. Table S3 shows the correlations between observed partners and swapped partners for covariates explaining individually more than 0.5 % of variation in height. (DOCX 1010 kb) [file 13059_2015_833_MOESM1_ESM.docx]

**Additional Figures**

**Fig. S1.** First (PCA1) and second (PCA2) principal components for phase 1 of the UK Biobank. Black colour indicates members of the couples with the classification of White-British, red colour indicates the rest of UK Biobank samples. The top figure includes all genotyped participants, whilst the bottom figure is a close-up to the area that includes the White-British.

| **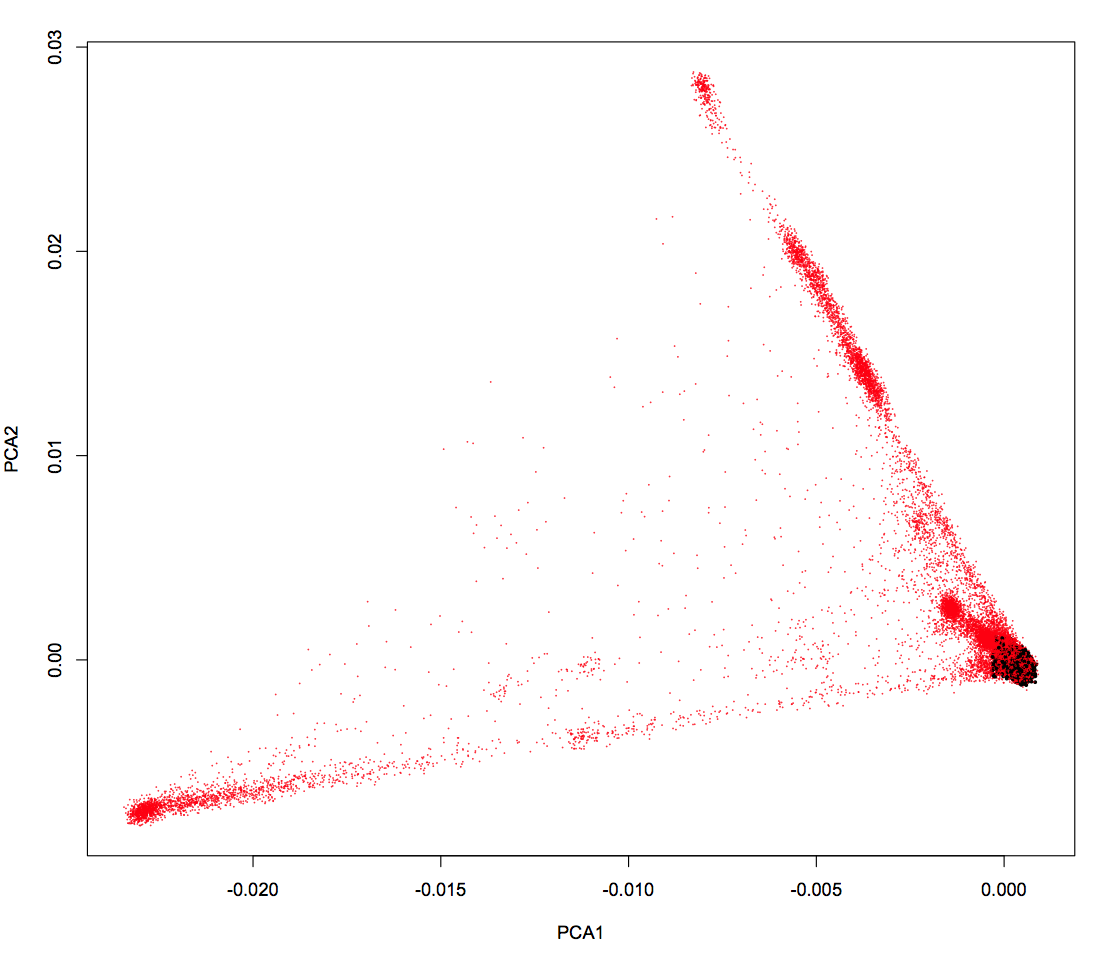** |
| --- |
|  |

**
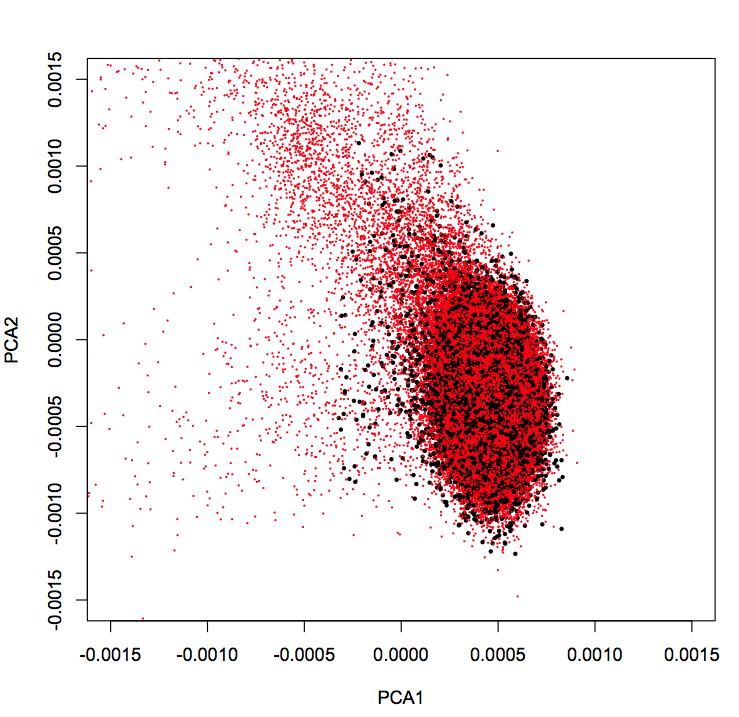
**

**Fig. S2.** Histogram of the relationships among couples.

**
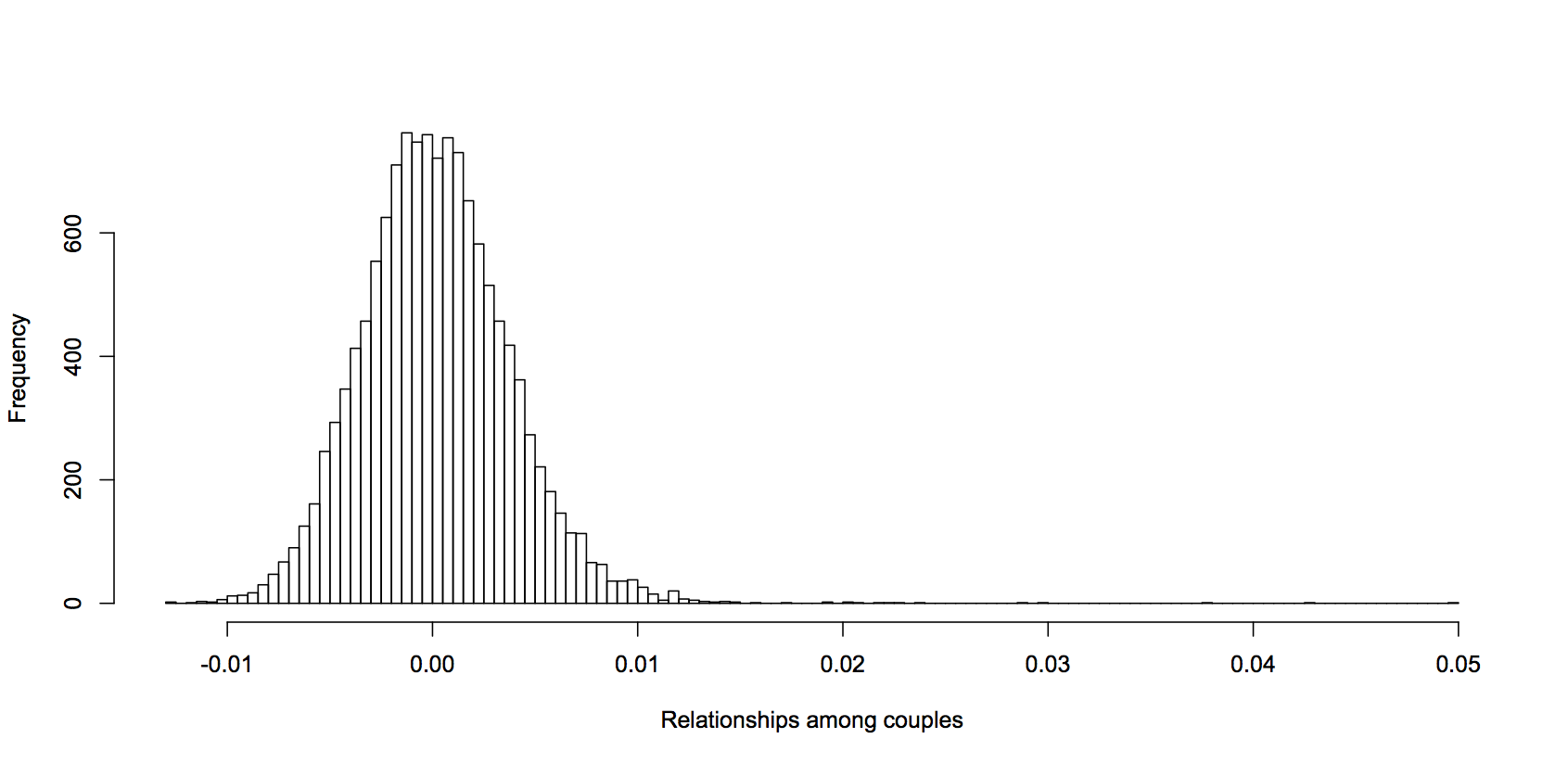
**

**Fig. S3.** Histogram of relationships used in the univariate and bivariate analyses. For display purposes, the left figure has r≤0.089 and the right figure has r>0.089.

**
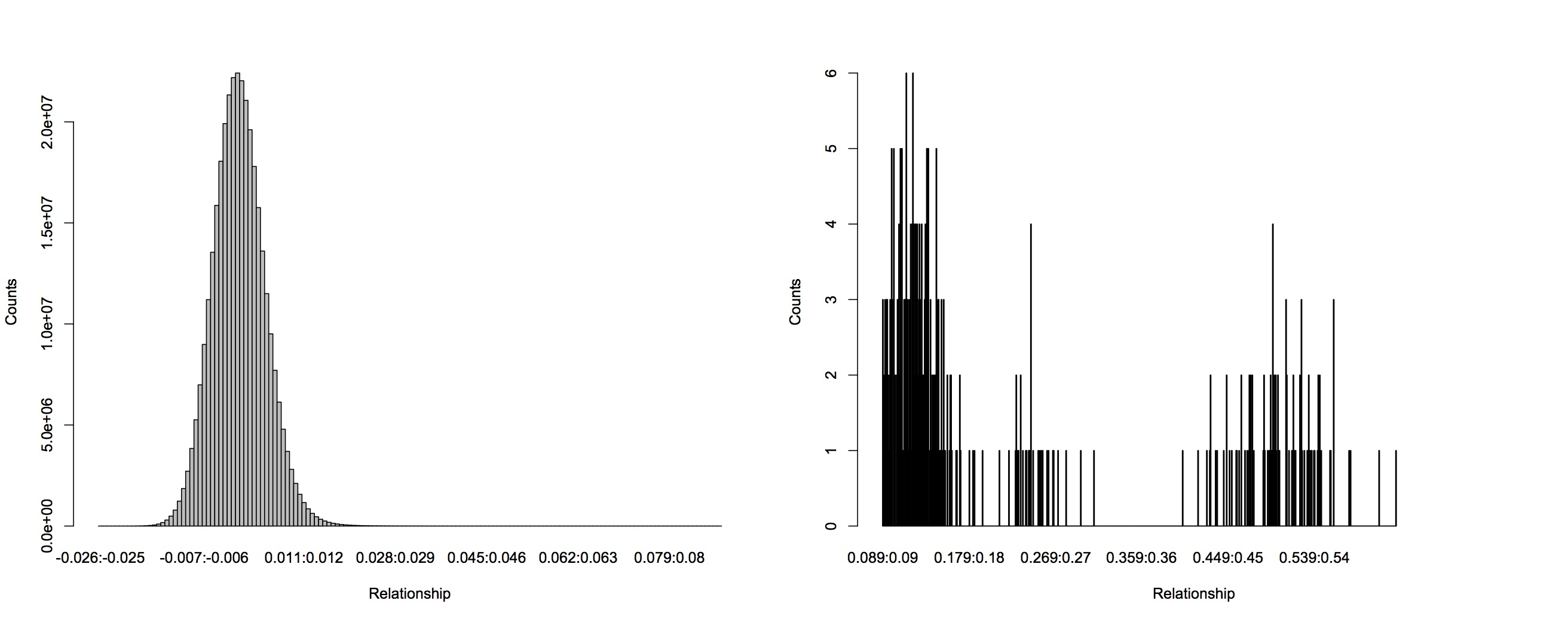
**

**Fig. S4.** Correlation of height (top figure) and correlation of height adjusted for gender, age, Townsend deprivation index and first 20 PCs.

| **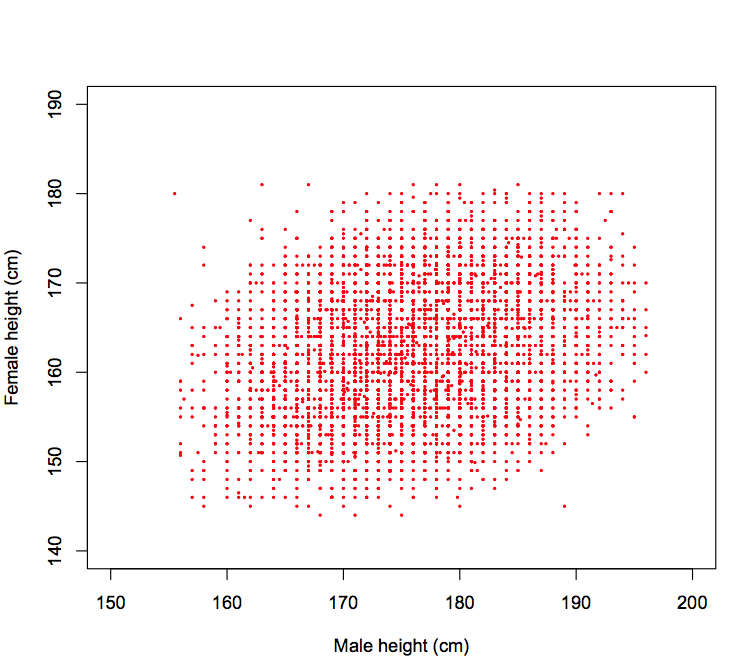** |
| --- |
|  |

**
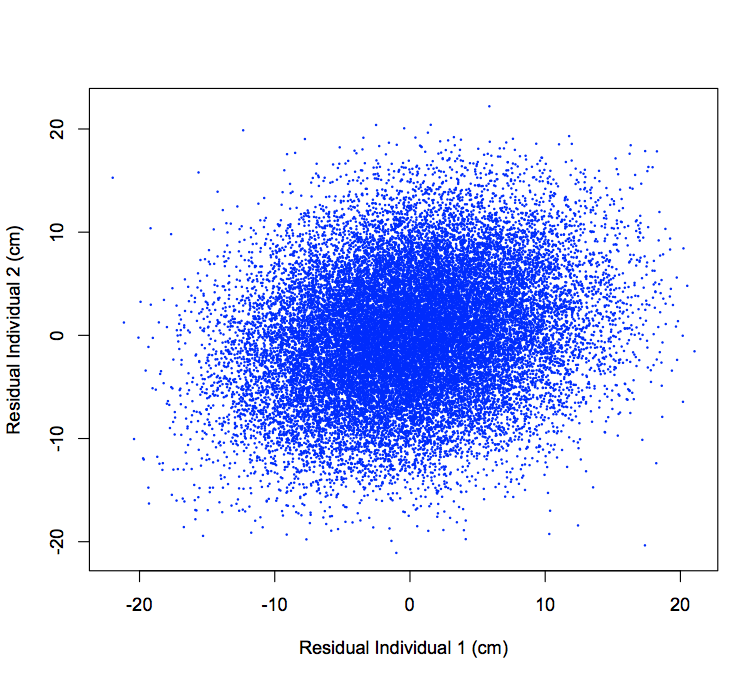
**

**Fig. S5.** Correlation among GWAS estimated effects for own height and partner’s height choice.

| **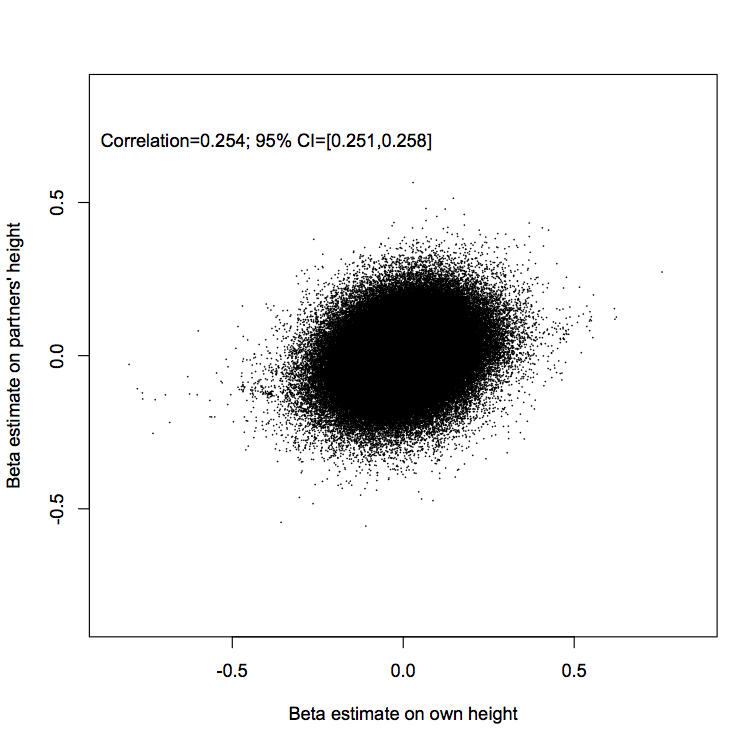** |
| --- |

**Additional Tables**

**Table S1.** Bivariate analysis of height (h^2^_Height_) and the choice of mate by height (h^2^_Height choice_) in mixed couples.

|  | Estimate | Standard Error |
| --- | --- | --- |
| *h^2^_Height_* | 0.619 | 0.052 |
| *h^2^_Height choice_* | 0.071 | 0.048 |
| *r_G_* | 0.286 | 0.178 |
| *r_E_* | 0.238 | 0.058 |
| *r_P_* | 0.201 | 0.011 |

**Table S2.** Bivariate analysis of height (h^2^_Height_) and choice of mate by height (h^2^_Height choice_) for White-British couples where partners have been swapped.

|  | Estimate | Standard Error |
| --- | --- | --- |
| *h^2^_Height_* | 0.599 | 0.015 |
| *h^2^_Height choice_* | 0.038 | 0.013 |
| *r_G_* | 0.893 | 0.142 |
| *r_E_* | 0.156 | 0.017 |
| *r_P_* | 0.231 | 0.006 |

**Table S3.** Correlations between observed partners and swapped partners for covariates explaining individually more than 0.5% of variation in height. For continuous covariates the Pearson’s correlation and two sided p values for the null hypothesis of zero correlation are given. For categorical variables, the mutual information and p values for the null hypothesis of zero mutual information are given.

|  | Covariate | Observed | Swapped |
| --- | --- | --- | --- |
| Continuous | Townsend deprivation index at recruitment | 1.00 (< 10^-100^) | 0.005 (0.54) |
|  | Age completed full time education | 0.16 (1.76x10^-34^) | 0.009 (0.5) |
|  | Birth weight | 0.04 (0.07) | -0.029 (0.17) |
|  | Place of birth in UK – north co-ordinate | 0.59 (< 10^-100^) | 0.015 (0.1) |
|  | Place of birth in UK – east co-ordinate | 0.35 (< 10^-100^) | -0.0004 (0.96) |
| Categorical | Drive faster than motorway speed limit | 0.0378 (< 0.001) | 0.0008 (0.17) |
|  | Average total household income before tax | 0.783 (< 0.001) | 0.0038 (< 0.001) |
|  | Job involves heavy manual or physical work | 0.012 (< 0.001) | 0.0007 (0.91) |
|  | Job involves mainly walking or standing | 0.007 (< 0.001) | 0.0008 (0.82) |
|  | Own or rent accommodation lived in | 0.648 (< 0.001) | 0.0021 (< 0.001) |
|  | Number of vehicles in household | 0.87 (< 0.001) | 0.0009 (0.1) |
|  | UK Biobank assessment centre | 2.791 (< 0.001) | 0.0214 (< 0.001) |
|  | Usual walking pace | 0.008 (< 0.001) | 0.0004 (0.05) |
